# Supplementary material for: Gait speed and incline modulate peak deceleration and timing of horizontal center of mass deceleration during double support
Source: Biol Open. 2025 Jul 7;14(7):bio062037. doi: 10.1242/bio.062037 (PMC12326370; doi:10.1242/bio.062037)
Supplement: Supplementary information [file biolopen-14-062037-s1.pdf]

Table S1. We conducted an exploratory subgroup analysis to examine potential sex-related differences in the main outcome variables (MD, MDt, SL, and DST). Independent-samples t-tests were performed for each variable under all four experimental conditions.

The results indicated no statistically significant differences between female and male participants for any of the variables across all conditions ($p > 0.10$ in all cases) (Appendix table).

		MD (m/s2)				MDt (%)			
		Level-moderate	level-fast	uphill-moderate	uphill-fast	Level-moderate	level-fast	uphill-moderate	uphill-fast
Male	Mean	5.28	5.23	3.33	3.43	63.69	63.61	62.78	63.31
	SD	0.93	0.63	0.60	0.82	1.78	1.30	1.75	2.46
Female	Mean	4.60	4.60	3.10	3.17	65.49	65.07	64.47	65.45
	SD	0.79	0.48	0.62	0.53	0.83	1.49	1.50	1.18
ttest	p value	0.26	0.09	0.56	0.59	0.11	0.09	0.14	0.16

		DST (%gait cycle)				SL (mm)			
		Level-moderate	level-fast	uphill-moderate	uphill-fast	Level-moderate	level-fast	uphill-moderate	uphill-fast
Male	Mean	7.97	7.81	23.66	6.07	463.73	528.26	514.98	559.74
	SD	2.15	1.03	38.45	3.55	27.54	31.52	85.42	88.13
Female	Mean	8.06	6.91	7.21	-4.58	455.90	501.97	481.07	528.32
	SD	1.88	1.99	0.99	30.69	28.88	19.62	33.25	36.57
ttest	p value	0.94	0.30	0.38	0.36	0.66	0.18	0.48	0.53

Biology Open • Supplementary information
